# Tubular atrophy/interstitial fibrosis scores of Oxford classification combinded with proteinuria level at biopsy provides earlier risk prediction in lgA nephropathy

**DOI:** 10.1038/s41598-017-01223-3

**Published:** 2017-04-24

**Authors:** Xuejing Zhu, Huiqiong Li, Yexin Liu, Jing You, Zhong Qu, Shuguang Yuan, Youming Peng, Fuyou Liu, Hong Liu

**Affiliations:** 1 Department of Nephrology, Second Xiangya Hospital, Central South University, Changsha, Hunan China; 2 Changsha Central Hospital, Changsha, Hunan China

## Abstract

The predictive effect of combining MEST with clinical data at biopsy on renal survival outcomes has not been investigated in patients with IgA nephropathy (IgAN). MEST of The Oxford classification of IgAN and 24-hour urine proteinuia measured at enrollment. The primary outcome was a composite of either ESRD (eGFR to <15 ml/min per 1.73 m^2^), or a permanent reduction in eGFR to below 50% of the value at biopsy. 742 patients were enrolled and follow-up >3 years, and were divided into two groups according to eGFR levels at biopsy. Multivariable logistical regression revealed that proteinuria at biopsy (OR 5.307 (95% Cl 3.003 to 9.376) p = 0.000), tubular atrophy/interstitial fibrosis scores (T) in MEST (OR 3.915 (95%Cl 2.710 to 5.654) p = 0.000) were the two predictors of eGFR decline for IgAN patients. Kaplan–Meier survival curves show significant difference in renal survival outcome among each T scores groups at biopsy (T0, T1, T2) (P < 0.05) and proteinuria levels at biopsy (P < 0.05), individially. Patients with T2 combined proteinuria at biopsy have the worst renal survival outcome. In conclusion, T scores in MEST classification combined with proteinuria at biopsy could be one of the important early predictors for the renal survial outcomes in patients with IgAN.

## Introduction

IgA nephropathy (IgAN) is one of the most common primary glomerulonephritis (GN), which is a main cause of ESRD in young adults^1, 2^. IgAN presents highly variable pathological and clinical changes from a very mild disease to end-stage renal disease (ESRD)^3^. Four types of histological lesions were identified by the MEST score of The Oxford classification of IgAN, including mesangial (M), endocapillary (E) hypercellularity, segmental sclerosis (S) and interstitial fibrosis/tubular atrophy (T)^4^. Although several studies have already focused on the prediction effect of MEST classificaiton, it remains unclear whether the clinical features combined with MEST score can improve the prediction of individual patient prognosis for their renal survial outcomes and guide management decisions at the time of biopsy.

Recently, several studies were trying to investigate the clinical or histological factors which could predict or determine the final outcome of patients with IgAN. It has been proved that time-averaged proteinuria during follow-up was the most important predictor of renal failure in IgAN^5^. Le W. *et al*. has reported that higher proteinuria, hypertension, impaired renal function, hypoproteinemia and hyperuricemia were independent predictors of an unfavorable renal outcome during the 20 years follow-up^6^. However, resent studies to determining the risk of renal progression in IgAN using clinical data alone is challenging owing to the highly variable pathological changes. In addition, long-time follow-up clinical data is needed before a meaningful prediction can be achieved in previous studies, which has has limited utility for the clinicians to make treatment decisions based on the clinical and pathological features at the early time of biopsy. Trying to identify the early predictor for the renal survial outcomes of IgAN patients at the time of biopsy seems to be more important.

The aim of this article is to investigate the prediction of renal outcome of IgAN patients using clinical variables with or without MEST classificaiton, in order to validate early clinical and pathological combinding predictors related to deterioration of renal function in IgAN patients.

## Methods

### Patients and study design

742 patients with biopsy-proven IgAN were enrolled in our hospital from 2008 to 2013, included patients with eGFR > 30 ml/min per 1.73 m^2^, and follow-up 3–8 years. Exclusion criteria were the renal transplantation, coexistence of other renal disease and the glomerulus in renal tissue less than 7 glomeruli. In addtion, we excluded those missing eGFR, blood pressure, and proteinuria at the time of biopsy. The eGFR was calculated using the Modification of Diet in Renal Disease (MDRD) study equation^7^.

This study was conducted in full adherence with the Declaration of Helsinki. Study protocols and all methods were approved by the Ethics Committee of Second Xiangya Hospital of Central South University. All subjects were informed of the protocols and gave their written consent prior to participating in the study.

### Renal biopsy and pathological classification

Renal biopsies were scored according to the MEST scoring system as part of the IgAN Oxford Classification by at least two pathological doctors confirmed the pathological results, who were all blinded to patient outcomes at the time of pathology review. Details of the histologic classification were as follows: M0 as amesangial score ≤0.5, M1 > 0.5, or ≤ or >50% of glomeruli with ≥4 mesangial cells per mesangial area, E0/E1 as the presence or absence of endocapillary hypercellularity, S0/S1 as the presence or absence of segmental sclerosis or tuft adhesions, and T0/T1/T2 as the degree of tubular atrophy or interstitial fibrosis (<25%, 25–50%, >50%, respectively)^4^.

End point Proteinuria, hemoglobine (Hb), mean artery pressure (MAP), albumin (ALB) and estimated glomerular filtration rate (eGFR) at biopsy were the closest values within 1 months of biopsy, whereas everage proteinuria levels were the average of all values over the at least 3 years after biopsy. The primary outcome was a composite of either ESRD (eGFR to <15 ml/min per 1.73 m^2^), or a permanent reduction in eGFR to below 50% of the value at biopsy. None of the patients received kidney transplantation during follow-up.

### Statistical analysis

Data were summarized as percentage or the mean (±standard deviation, SD) as appropriate. Fisher’s exact test or Chi-square test were used to analysis the categorical variables and continuous variables were compared by using t-test, Mann-Whitney U-test, Kruskal-Wallis H-test. Patients were divided depending on eGFR levels at biopsy. Spearman’s correlation was used to evaluate the correlations among each histopathological and clinical finding. Here we used the coefficient (B) and odds ratio (OR = EXP (B)) along with its 95% confidence interval (95%CI) to reflect the dependent variable to elevate one or more levels, in response to the independent variable changing for every one unite. Cumulative survival was estimated with Kaplan–Meier survival curves, and was compared by using the log-rank test. Five models were created: (i) patients were grouped by T scores (T0, T1, T2); (ii) grouped by different proteinuria levels at biosy (0, 0–3.5 g/24 hours, >3.5 g/24 hours); (iii) grouped by T scores combined different proteinuria levels at biosy (T0-1 or T2 with proteinuria levels <150 or ≥150 mg/24 h);(iv) grouped by T scores combined with proteinuria levels ≥ or <1.0 g/24 h; (v) grouped by T scores combined with proteinuria levels ≥ or <2.0 g/24 h. A P-value of <0.05 was regarded as statistical significance. SPSS 17.0 was used to store and analysis our data.

## Results

### Clinical findings among different eGFR levels at biopsy time

Of the 742 patients, 328 were men (44.2%). The mean eGFR was 82.7 ± 1.0 ml/min/1.73 m^2^. Urinary protein excretion was 1.5 ± 0.1 g/day. Clinical findings among different eGFR levels (group 1: eGFR ≥ 60 ml/min/1.73 m^2^, group 2:eGFR < 60 ml/min/1.73 m^2^) are displayed in Table 1. In group 2 (eGFR < 60 ml/min/1.73 m^2^), the patients have significantly higher age, urine protein excrecion, MAP level, s-Cr, CHOL, serum IgA/IgG ratio and serum C4 levels, compared to the patients with eGFR ≥ 60 ml/min/1.73 m^2^ (p < 0.05), and have lower levels of s-Alb and Hb (p < 0.05). Treatment including immunosuppression or renin-angiotensin system blockade (RASB) during follow-up was used in 39.1% in group1 and 49.1% in group 2 (p < 0.05). In order to identify whether the treatments will influence the statistical results, we divided cases into two subgroups depending on the cases who did get and who did not get the treatment in each groups (eGFR ≥ 60 or <60 ml/min/1.73 m^2^). In the group eGFR ≥ 60 ml/min/1.73 m^2^, there was no statistical significance for age (p = 0.897), Hb (p = 0.963), M (p = 0.283), E (p = 0.651), S (p = 524), T (p = 0.928), proteinuria level at biopsy time (p = 0.172) between two subgroups. In the group eGFR < 60 ml/min/1.73 m^2^, there was also no statistical significance for age (p = 0.311), Hb (p = 0.714), M (p = 0.141), E (p = 0.441), S (p = 0.387), T (p = 0.102), proteinuria level at biopsy time (p = 0.276) between two subgroups.

**Table 1:** Clinical finding among different eGFR levels at biopsy time.

	All (n = 742)	eGFR ≥ 60 (n = 632)	eGFR < 60 (n = 110)
Age(year)	32.7 ± 0.5	31.3 ± 0.4	41.6 ± 1.3*
U-Pro(g/day)	1.5 ± 0.1	1.3 ± 0.1	2.8 ± 0.3*
MAP(mmHg)	95.0 ± 0.5	93.8 ± 0.5	101.6 ± 1.3*
s-Cr(umol/l)	84.9 ± 1.3	74.8 ± 0.9	145.2 ± 4.1*
BUN(mmol/l)	8.6 ± 1.2	8.5 ± 1.4	9.0 ± 0.4
UA(umol/l)	340.5 ± 4.0	327.5 ± 4.0	419.4 ± 12.1
s-Alb(g/dl)	36.1 ± 0.3	36.8 ± 0.3	31.9 ± 1.0*
Hb(g/dl)	127.6 ± 2.1	134.7 ± 3.6	122.2 ± 2.4*
CHOL(mmol/l)	5.5 ± 0.1	5.4 ± 0.1	6.1 ± 0.2*
TG(mmol/l)	1.9 ± 0.1	1.8 ± 0.1	2.1 ± 0.1
IgG(g/l)	10.2 ± 0.2	10.4 ± 0.2	9.5 ± 0.5
IgA(g/l)	2.6 ± 0.0	2.5 ± 0.0	2.8 ± 0.1*
IgA/C3	2.6 ± 0.1	2.6 ± 0.1	2.8 ± 0.1
IgM(g/l)	1.9 ± 0.5	2.0 ± 0.6	1.4 ± 0.1
C3(g/l)	1.0 ± 0.0	1.0 ± 0.0	1.0 ± 0.0
IgA/IgG	0.3 ± 0.0	0.3 ± 0.0	0.5 ± 0.1*
C4(g/l)	0.2 ± 0.0	0.2 ± 0.0	0.3 ± 0.0*
Treatment	301(40.6%)	247(39.1%)	54(49.1%)*

U-Pro, urinary protein excretion; MAP, mean artery pressure; s-Cr, serum creatinine; BUN, blood urea nitrogen; UA, uric acid; s-Alb, serum albumin; Hb, hemoglobine; CHOL, cholesterol; TG, triglyceride; Treatment: Use of ARSB or any immonosuppression; *p < 0.05 compared to eGFR ≥ 60 group.

### Correlations among the clinical findings among different eGFR levels at biopsy time

Correlations among the clinical findings at biopsy time and MEST scores are analyzed. The eGFR showed positive correlation with urinary protein excretion (r = 0.229, p = 0.000), and tubular atrophy/interstitial fibrosis scores (T scores) (r = 0.329, p = 0.000). The eGFR also showed positive correlation with M score (r = 0.092, p = 0.012), S score (r = 0.110, p = 0.003) and treatment (r = 0.077, p = 0.035), but r values were relatively lower. There was no statistical significant correlation between eGFR and age (p = 0.098), Hb (p = 0.067), ALB (p = 0.097), E score (p = 0.543). Double serum creatinine at the end of 3 years showed positive correlation with T score (r = 1.184, p = 0.011).

We also analyzed the every subgroup patients who did get or did not get the treatment in the group eGFR ≥ 60, 45–59, and <45 ml/min/1.73 m^2^ separately. There was no statistical significant correlation between treatment and M (p = 0.283), E (p = 0.651), S (p = 0.524), T scores (p = 0.938) in the patients who eGFR ≥ 60 ml/min/1.73 m^2^; M (p = 0.228), E (p = 0.860), S (p = 0.938), T scores (p = 0.667) in the patients who eGFR 45–59 ml/min/1.73 m^2^; M (p = 0.281), E (p = 0.390), S (p = 0.244), T scores (p = 0.060) in the patients who eGFR < 45 ml/min/1.73 m^2^.

Correlations among the MEST scores and urinary protein excretion at biopsy time are also analyzed. T scores showed positive correlation with proteinuria >1 g/d (r = 0.301, p = 0.000), but no statistical significant correlation with proteinuria >2 g/d (p = 0.354). S scores showed positive correlation with proteinuria >1 g/d (r = 0.190, p = 0.015). There are no statistical significant correlation between proteinuria >1 g/d with M scores (p = 0.550) and E scores (p = 0.142), and no statistical significant correlation between proteinuria >2 g/d with M scores (p = 0.304), E scores (p = 0.570) and S scores (p = 0.226).

### Multivariable logistical regression among different eGFR levels at biopsy time

Multivariable logistical regression revealed that urinary protein excretion ((OR 5.307 (95%Cl 3.003 to 9.376) p = 0.000), tubular atrophy/interstitial fibrosis scores in MEST ((OR 3.915 (95%Cl 2.710 to 5.654) p = 0.000) were associated with eGFR (Table 2). There was no statistical significance between eGFR with M, E, S scores (p = 0.158, 0.857, 0.936) and treatment (p = 0.104).

**Table 2:** The Multivariable logistical regression at the time of renal biopsy.

	OR	Std. Error	Wald	Sig.	95% Confidence Interval	
					Lower Bound	Upper Bound
M	1.819	0.424	1.990	0.158	0.792	4.177
E	1.067	0.359	0.032	0.857	0.527	2.157
S	0.994	0.076	0.006	0.936	0.856	1.154
T	5.307	0.188	52.926	0.000	2.710	5.654
U-Pro	3.915	0.290	33.022	0.000	3.003	9.376
Treatment	1.518	0.257	2.645	0.104	0.918	2.511

M, Mesangial hypercellularity, E, Endocapillary hypercellularity, S, Segmental glomerulosclerosis, T, Tubular atrophy/interstitial fibrosis, U-Pro, urinary protein excretion; Treatment:Use of ARBs or any immunosuppression.

### Multivariable logistical regression among different eGFR levels during follow-up periods

Containing at least 3-year clinical data, results of the Multivariable logistical regression revealed that tubular atrophy/interstitial fibrosis scores in MEST (OR 3.488 (95%Cl 1.770 to 6.875) p = 0.000) were associated with eGFR. There was no statistical significance between eGFR with everage urinary proteinuria (p = 0.081), M (p = 0.094), E (p = 0.463), S scores (p = 0.191). Double serum creatinine at the end of 3 years were associated with T scores (OR 2.022 (95%Cl 1.114 to 3.670) p = 0.021). There was no statistical significant between double serum creatinine and M (p = 0.223), E (p = 0.794), S scores (p = 0.274).

### Kaplan–Meier survival curves on different T scores or/with proteinuria levels at biopsy time

**Figure 1:**
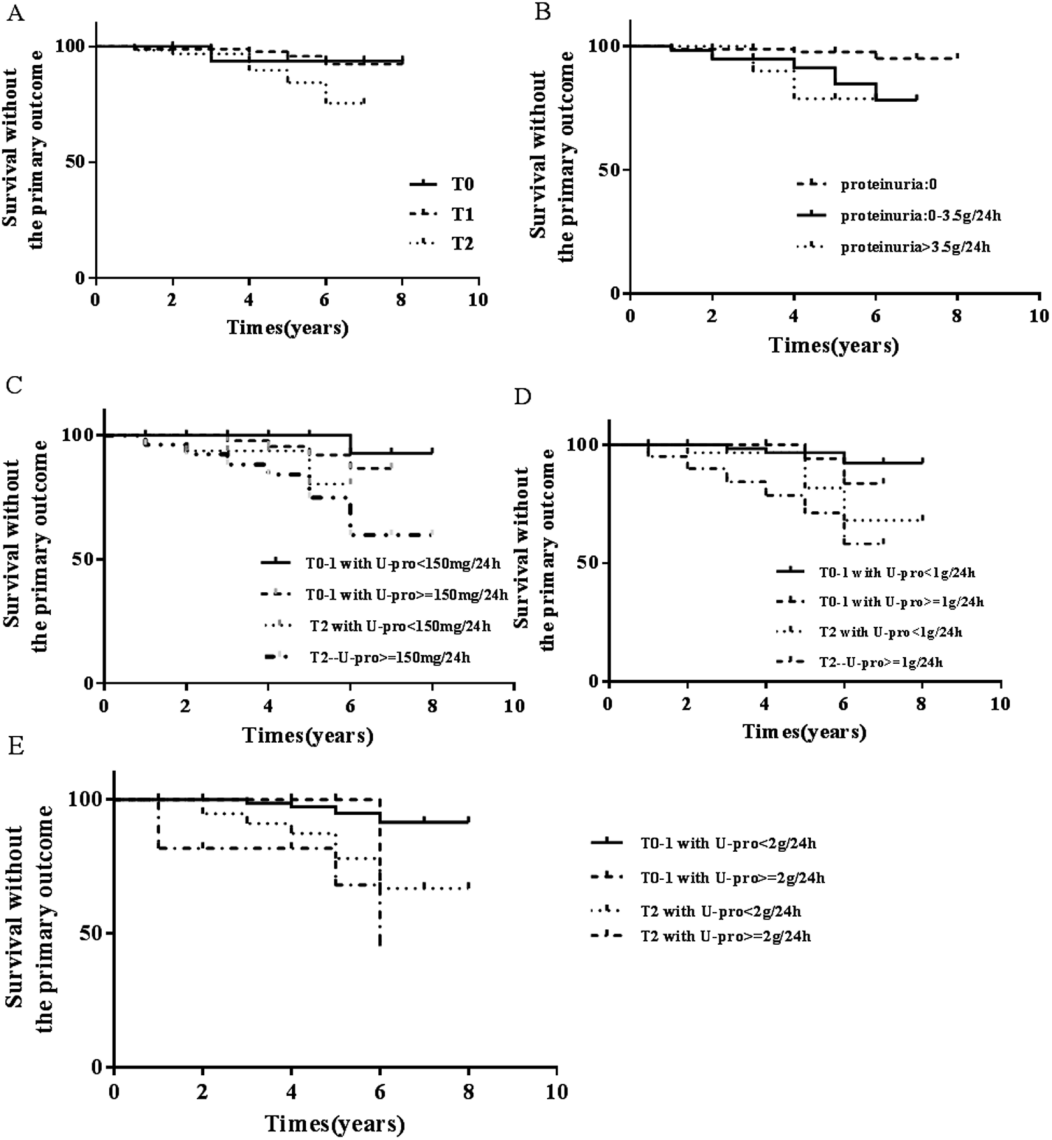
The risk of the primary renal outcome (50% reduction in estimated eGFR or endstage renal disease) according to T scores and proteinuria. Kaplan–Meier survival curves stratified according to the scores for tubular atrophy/interstitial fibrosis (**A**), proteinuria levels at the time of biopsy (**B**), different T scores combinded with/without proteinuria at biopsy (**C**), different T scores combinded with proteinuria levels ≥ or <1.0 g/24 h (**D**), and different T scores combinded with proteinuria levels ≥ or <2.0 g/24 h (**E**).

**Figure 2:**
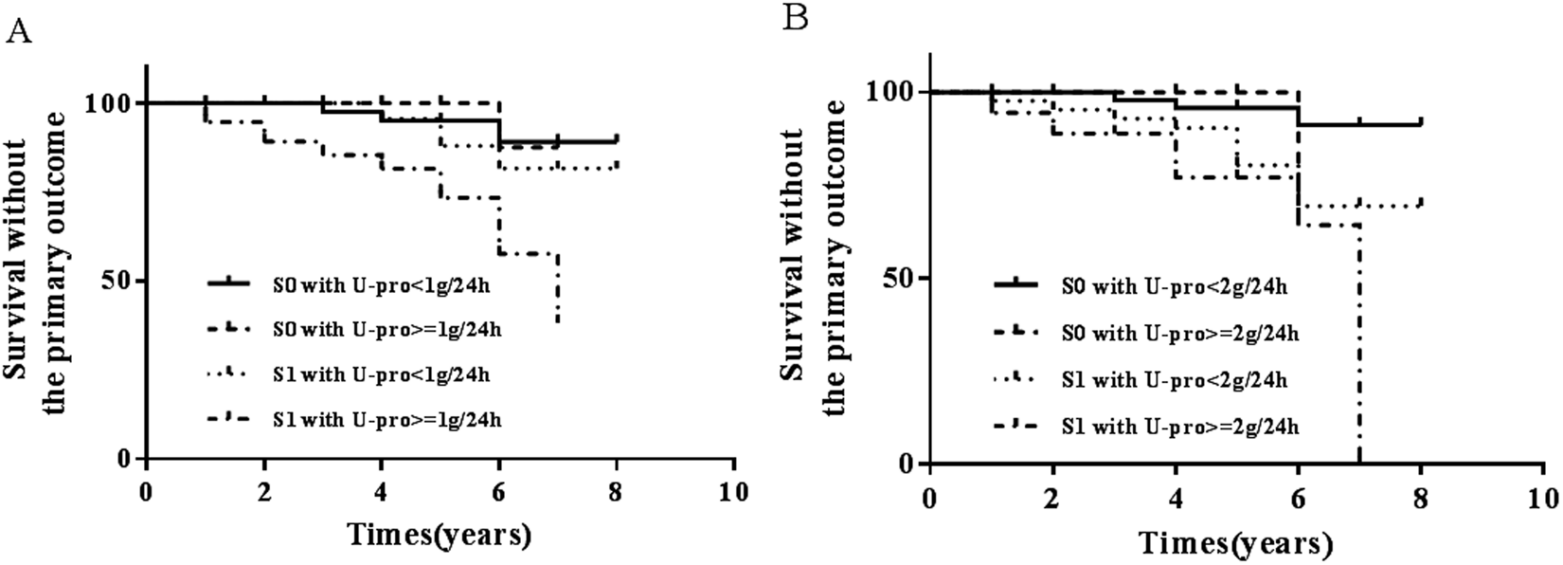
Kaplan–Meier survival curves according to segmental glomerulosclerosis. Kaplan–Meier survival curves stratified according to the scores for segmental glomerulosclerosis combinded with proteinuria levels ≥ or <1.0 g/24 h at biopsy (**A**), and different T scores combinded with proteinuria levels≥ or <2.0 g/24 h at biopsy (**B**).

Kaplan–Meier survival curves stratified according to the scores for tubular atrophy/interstitial fibrosis (Fig. 1A) or proteinuria levels at the time of biopsy (Fig. 1B). There was a significant in renal survival among each tubular atrophy/interstitial fibrosis scores groups (P < 0.05) (Log-rank test shows p value = 0.047) and proteinuria levels groups (P < 0.05) (Log-rank test shows p value = 0.022). We generated subgroups with different T scores combined with/without proteinuria at biopsy whose initial eGFR was >60 ml/min/1.73 m^2^ (Fig. 1C). Patients with tubular atrophy/interstitial fibrosis T2 and proteinuria at biopsy have the worst renal survival outcome, compared with other groups (Log-rank test shows p value = 0.003). The renal outcome was much better in patients with T0 and T1 with/without proteinuria at biopsy, compared with the patients with T2 (p < 0.01). And we also generated subgroups with different T scores combined with/without proteinuria ≥1 g/24 h (Fig. 1D) or 2 g/d (Fig. 1E) at biopsy. Patients with T2 and proteinuria ≥1 g/24 h (Log-rank test shows p value = 0.002) or 2 g/24 h (Log-rank test shows p value = 0.010) at biopsy have the worst renal survival outcome.

In addition, we generated subgroups with different M, E, S scores combined with/without proteinuria ≥1 g/24hor 2 g/24 h at biopsy. Kaplan–Meier survival curves show S1 with proteinuria ≥1 g/24 h **(**Fig. 2A) and ≥2 g/24 h **(**Fig. 2B **)** impaired renal survivial (Log-rank test shows p value = 0.001, 0.029). On the contrary, M and E scores was not significantly associated with renal outcome (Log-rank test shows p value = 0.108, 0.066 respectively with proteinuria ≥1 g/24 h; p value = 0.478, 0.098 with proteinuria ≥2 g/24 h).

## Discussion

In this study, we validated tubular atrophy/interstitial fibrosis scores and proteinuria levels at the time of renal biopsy, especially T2 combined with proteinuria, could be the important risk factors for the decrease of eGFR in patients with IgAN, which were the early pathological and clinical predictors for the renal survival outcomes for IgAN patients.

Previous Lee histology classification was identified insufficiently to predict the decline of renal function, and long-time follow-up of proteinuria dectection was required^8, 9^, which is limited for the early effective treatment and prediction in order to improve the preservation of renal function and potentially delay the progression of IgAN. We investigated the correlations among the clinical findings and MEST scores in different levels of baseline eGFR. Results indicatied that decreased eGFR were significantly related to proteinuria, M, S and T scorses at the time of renal biopsy. T scores show strong correlation with proteinuria levels at biopsy time and double serum creatinine at the end of 3 years. S scores also show relatively weaker correlation with the proteinuria levels. In clinical data, further regression analysis demonstrated that only proteinuria level at the time of biopsy, but not the everage proteinuria level during follow-up period, could be an effective predictor for eGFR decline in IgAN patients.

In addtion, our present study provides novel insights into the importance of tubular atrophy/interstitial fibrosis levels in Oxford Classification of IgAN patients. Several studies demonstrate a clear association between mesangial hypercellularity and renal outcome at biopsy or over time^10^. An analysis of 261 cases from the VALIGA European cohort have reported that patients with M1 were at risk of developing higher time-averaged proteinuria, indicating the predictive effect of M on young patients with IgAN^11^, However, there have been less consistent findings with tubular atrophy/interstitial fibrosis levels. In this study, we demonstrate that T scores in MEST classification was at risk of developing eGFR decline in regression analysis at baseline time or during follow-up and also of double serum creatinine during follow-up, indicating that T1-2 score was associated with the composite renal outcome independent of clinical data at biopsy or over time. This suggests that both proteinuria level and tubular atrophy/interstitial fibrosis levels at biopsy time could provide earlier and better predictions of the patient-level risk of a 50% decline in renal function or ESRD, which means there are independently important predictors for the progression of nephron function in IgAN patients.

More and more studies have reported the some or all of the MEST components and several clinical features as the independent predictors for IgAN patients since the publication of original MEST Oxford Classification. Tanaka S *et al*. have identified proteinuria, estimated GFR, mesangial proliferation, segmental sclerosis, and interstitial fibrosis/tubular atrophy (T2: HR, 20.5; 95%CI, 9.05 to 46.5) as independent risk factors for developing ESRD^12^. However, the association between tubular atrophy/interstitial fibrosis (T) scores combined with proteinuria at the time of biopsy and renal outcome during follow-up have not reported yet. In this study, we are trying to use more detailed analysis in order to figure out more accurate risk factor for renal outcome. Our results show patients with tubular atrophy/interstitial fibrosis T2 and proteinuria at biopsy have the worst renal prognosis, patients with T2 but without proteinuria have a similar renal prognosis as those with T2 and proteinuria, indicating that despite lower protein excretion, T2 is considered to be high-risk factor for the bad renal survial prognosis. Conversely, patients with proteinuria at biopsy but without T2 have a relatively favorable prognosis, suggesting proteinuria at biopsy is a relative weaker risk factor compared to T2. These results indicatied that combining the MEST score (T2) with patient’s clinical data (proteinuria) at biopsy will provide significant improvement for earlier and more accurate risk prediction in IgAN. In addition, segmental glomerulosclerosis also worsened the renal outcome when combined with proteinuria at the time of biopsy, indicating chronic glomerular injury might influence the renal survival prognosis.

There were several limitations in this study. First, the number of cases in group eGFR 30–60 ml/min/1.73 m^2^ was relatively small, which might influent the results. For the patients with lower eGFR would not tend to be performed with renal biopsy examination. Secondly, in our results, there was no statistical significance correlation between eGFR with average urinary proteinuria furing follow-up. However, previous study found that average urinary proteinuria is a predictor of renal prognosis^13^. This might because several patients in this study were performed urine routine detection instead of 24 hour- proteinuria data during follow-up period, resulting in that data of average urinary proteinuria contained only two time point in a part of patients, which would weaken the association found in this study.

In conclusion, we have demostrated MEST (especially T2) combined with proteinuria at biopsy could be a strong signal for the bad renal survival outcomes in patients with IgAN. These results illustrations demonstrate the potential benefits for the clinicians to make early decisions and judgements from accurate risk prediction at the time of biopsy using the MEST score.
